# Investigating the impact of dual-chain coupling on new quality productive forces in leading traditional Chinese medicine enterprises

**DOI:** 10.3389/fmed.2026.1828456

**Published:** 2026-05-25

**Authors:** Zhiguang Li, Wenwu Zhao, Fan Zhang, Ruijin Xie

**Affiliations:** 1 School of Economics and Management, Anhui University of Chinese Medicine, Hefei, China; 2 Key Laboratory of Data Science and Innovative Development of Traditional Chinese Medicine, Philosophy and Social Sciences of Anhui Province, Hefei, China

**Keywords:** dual-chain synergy, innovation investment, new quality productive forces, public health, supply chain, talent chain, traditional Chinese medicine

## Abstract

**Background:**

New quality productive forces are increasingly recognized as essential drivers of high-quality development in health-related industries. In the context of TCM, the synergy between talent chain and supply chain may play a pivotal role in shaping firms’ capacity to contribute to public health goals.

**Purpose:**

This study investigates the impact of talent-supply chain synergy on new quality productive forces in leading TCM manufacturing enterprises, and examines the mediating role of innovation investment. It also explores how regional institutional contexts, such as TCM healthcare infrastructure, education systems, and policy environments, moderate this relationship.

**Methods:**

The study utilizes panel data from 75 publicly listed TCM manufacturers in China from 2015 to 2023. A stepwise regression is conducted to assess the effects of talent-supply chain synergy on new quality productive forces and the mediating role of innovation investment. In addition, heterogeneity across regional institutional contexts, including TCM healthcare infrastructure, education systems, and policy environments, is to examine the effects of dual-chain coupling coordination vary under different contextual conditions.

**Findings:**

The results indicate that: (1) the coupled synergy between the talent chain and the supply chain significantly enhances firms’ new quality productive forces; (2) innovation investment plays a critical mediating role in linking dual-chain synergy to the development of new quality productive forces; and (3) the positive effects of dual-chain synergy are more pronounced in regions with stronger TCM healthcare infrastructure, more developed TCM education systems, and more favorable policy environments.

**Originality/value:**

This study offers theoretical insights into how dual-chain coordination contributes to the advancement of new quality productive forces and provides practical guidance for TCM enterprises in enhancing innovation investment and optimizing their alignment with regional institutional contexts.

## 1 Introduction

In September 2023, during an inspection tour in Heilongjiang Province, General Secretary Xi Jinping emphasized the need to integrate scientific and technological innovation resources, lead the development of strategic emerging and future industries, and accelerate the formation of new quality productive forces (1). The report of the *20th National Congress of the Communist Party of China* further called for promoting the inheritance and innovation of traditional Chinese medicine (TCM), while the *Third Plenary Session of the 20th Central Committee* proposed strengthening support mechanisms for innovative drugs and improving related policy frameworks. These directives reflect the state’s strategic emphasis on technological innovation and provide clear guidance for industrial upgrading, particularly in the TCM sector. As a field with strong indigenous intellectual property and deep cultural roots, the TCM industry holds considerable potential for innovation (2–4). Listed TCM enterprises, as core actors linking upstream and downstream segments and integrating internal and external resources, play a crucial role in preserving traditional knowledge while promoting innovation, thereby driving the modernization and industrialization of traditional Chinese medicine (5).

In recent years, scholarly attention to the modernization of the TCM industry has deepened, particularly concerning how to foster the development of firms’ new quality productive forces (NQPF) within this modernization process (6). Existing research mainly adopts a macro perspective to analyze the opportunities and challenges faced by TCM manufacturing firms, as well as the pathways to achieving innovation-driven growth under policy guidance (7–9). Studies on the development of NQPF have focused on factor-driven mechanisms, corporate governance, and government-led digital governance. From the factor-driven perspective, scholars explore how emerging digital inputs, such as data elements, empower firms’ NQPF (10). At the corporate governance level, advances in ESG (11), artificial intelligence (12), and digital governance (13) are recognized as critical drivers of NQPF development. However, the rising cost of equity capital may impede these efforts by limiting R&D investment and undermining governance quality, thereby posing a potential constraint on NQPF enhancement (14).

Although NQPF are widely recognized as key drivers of economic and social development (15–17), a systematic definition, delineation of content, and identification of constitutive elements remain underdeveloped within the specific context of TCM manufacturing firms. This gap limits a deeper understanding of emerging drivers in the TCM industry, particularly with respect to the practical guidance for identifying and leveraging critical, disruptive technological breakthroughs. Furthermore, existing studies that examine the determinants influencing the development of NQPF tend to concentrate on artificial intelligence, big data applications, digital transformation, and innovative resource allocation (18–21), while comparatively little attention has been paid to the synergistic interactions along internal and external firm chains, in particular the coupling and collaboration between talent chains and supply chains, which play a crucial role in driving NQPF.

Building on this, the study investigates the impact of the coupled synergy between the talent chain and the supply chain on NQPF in leading TCM manufacturers. Compared with prior research, this study makes three key contributions. First, it conceptualizes the coupling and coordination of the talent and supply chains as the central analytical perspective, examining how their interaction effectively promotes firms’ NQPF. This dual-chain approach not only extends beyond traditional single-chain frameworks but also addresses the existing gap regarding the role of dual-chain synergy in enhancing NQPF within the TCM industry. Second, the study delves into the mediating role of innovation investment in the relationship between dual-chain synergy and NQPF, revealing the mechanism through which coordinated development of these two chains fosters the enhancement of firms’ productive capabilities. This provides a novel theoretical framework for understanding innovation investment as a conduit for firm development. Third, the research incorporates regional heterogeneity by exploring how differences in TCM healthcare infrastructure, educational systems, and policy environments moderate the effectiveness of dual-chain synergy on NQPF.

## 2 Literature review and research hypotheses

### 2.1 Talent chain and firms’ new quality productive forces

Talent serves as a fundamental and strategic support for the comprehensive modernization of socialist countries and plays a critical role in the development of NQPF in China. Centered on technological innovation as the core driver, NQPF requires firms to possess strong innovative capabilities and core competitiveness, both of which depend heavily on the intellectual support and innovation leadership provided by diverse high-quality talent pools (22, 23). Theoretically, the four-chain integration framework emphasizes that the deep integration of the innovation chain, industrial chain, capital chain, and talent chain is a key pathway to promoting NQPF development (24). Among these, the talent chain acts as the critical element supplying human resources to the innovation and industrial chains, thereby constituting the core of the entire system. In the process of technological innovation, high-level R&D talent is essential for overcoming critical technological bottlenecks and creating firm’s core competitiveness (25). Meanwhile, technical specialists play a pivotal role in translating research outcomes into productive capacity, thereby enhancing firms’ operational efficiency and product quality (26). In addition, managerial talent contributes by optimizing organizational structures and operational models, which improves management effectiveness and market competitiveness (27).

From a practical perspective, numerous studies have emphasized the critical role of the talent chain across various industries and sectors (28). For instance, in the artificial intelligence industry, the effective integration of the innovation chain and the talent chain has been shown to inject significant momentum into industry development (29). Moreover, the coupled development of the talent chain and industrial chain facilitates the restructuring of industrial linkages, thereby promoting high-quality regional collaborative development (30). These findings collectively underscore the close connection between the talent chain and industrial development, as well as their positive impact on enhancing industrial productivity. At the firm level, possessing a highly skilled and specialized workforce is crucial for gaining a competitive advantage in a fiercely contested market. In the context of traditional Chinese medicine, the influence of human capital on systemic coordinated development has attracted scholarly attention. Drawing on provincial-level panel data from China, it has been found that the level of TCM human capital significantly affects the coupling coordination degree between medical services and economic development, and that optimizing the regional allocation of medical personnel is a key pathway to enhancing systemic synergy (31). By attracting and cultivating talent aligned with the demands of new quality productive forces, firms are better equipped to respond to market shifts and technological transformations, thus enabling innovation-driven growth (32). Based on this, the following hypothesis is proposed:

*H1*: The talent chain has a significant positive effect on the development of firms’ new quality productive forces.

### 2.2 Supply chain and firms’ new quality productive forces

In the advancement of NQPF, the supply chain plays an equally critical role alongside the talent chain. Acting as a bridge connecting internal and external resources, the supply chain exerts a significant influence on the development of NQPF (33). By optimizing their supply chains, firms can achieve efficient allocation of resources both within and beyond organizational boundaries. Through the absorption and sharing of knowledge and technology, firms are able to engage in collaborative innovation, thereby effectively driving the development of NQPF (34). Specifically, firms can realize qualitative leaps in productivity by optimizing production processes, improving operational efficiency, and reducing costs. Such progress extends beyond the automation and intelligence of physical production to encompass knowledge creation and innovation in service industries (35). From a smart supply chain perspective, enterprises leverage technological exchange and knowledge sharing to strengthen bilateral or multilateral learning among members, enhancing the effectiveness of such learning and jointly promoting technological innovation and industrial upgrading (36). The resultant digital and intelligent transformation driven by industrial upgrading provides resource and structural empowerment, thereby significantly enhancing manufacturing firms’ NQPF.

From a production and service standpoint, firms with strong supply chain integration capabilities can generate notable factor allocation effects that boost technological innovation capacity while fostering governance effects that improve operational efficiency and reduce external dependency, thus enhancing productivity (37). In the traditional Chinese medicine sector, the unique challenges of raw material authenticity and quality control underscore the critical importance of supply chain governance (38). Furthermore, such firms are better positioned to respond rapidly to market changes, meet consumer demands flexibly, and adjust pricing strategies to manage costs and capture market share, ultimately securing competitive advantages (39). Fundamentally, supply chain capability manifests not only in superior resource utilization efficiency during value creation compared to competitors but also in value creation outcomes, such as significant advantages in cost, quality, and consistency (40). These advantages help firms maintain resource scarcity, inimitability, and non-substitutability. Conversely, firms with underdeveloped supply chains face multiple challenges. Lacking upstream and downstream connectivity, such firms struggle to sustain learning and assimilate the latest external technologies and market knowledge, hindering adaptation to rapidly evolving market environments. Additionally, poor internal supply management leads to rising production costs and shrinking profit margins, ultimately resulting in loss of competitive position. In summary, firms with well-developed supply chains are better equipped to continuously enhance their innovation capabilities and competitiveness, thereby improving economic and productive efficiency. Based on these considerations, the following hypothesis is proposed:

*H2*: The supply chain has a significant positive effect on the development of firms’ new quality productive forces.

### 2.3 Coupled and coordinated development of the dual chains and firms’ new quality productive forces

The coupled and coordinated development of the talent chain and supply chain refers to a dynamic interactive process in which the two chains form a synergistic relationship characterized by positive interaction and mutual reinforcement (41). In the current market environment, human capital acts as the intellectual foundation for innovation, transformation, and industrial upgrading, with talent, composed of critical cognitive elements such as knowledge, skills, and competencies, serving as the core driver of firm development (42). When a well-matched talent pool is integrated into various segments and departments of a firm’s supply chain, it can directly enhance industrial efficiency through knowledge spillovers and complementary advantages with other production factors. This process plays a crucial role in strengthening organizational resilience and ensuring operational security. Conversely, the supply chain holds unique value for talent management and development within firms. By facilitating comprehensive talent audits, the supply chain enables firms to accurately identify gaps between existing talent stocks and actual demand, thereby allowing targeted talent cultivation to ensure an adequate supply of professionals aligned with business development needs (43). Thus, the talent chain and supply chain are mutually dependent and promotive, jointly generating synergistic effects.

Firms equipped with robust supply chain systems and sufficient talent reserves can flexibly adjust internal production arrangements and market response strategies. Faced with market uncertainties, such firms can leverage innovative solutions and advanced technologies to optimize resource allocation and utilization, thereby continuously enhancing their NQPF. Consequently, the coordinated development of the talent and supply chains not only improves firms’ innovation capabilities and resource allocation efficiency but also strengthens their competitiveness and sustainability, significantly contributing to the cultivation and enhancement of NQPF. Based on these considerations, the following hypothesis is proposed:

*H3:* The coupled and coordinated development of the talent chain and supply chain has a significant positive effect on the development of firms’ new quality productive forces.

### 2.4 Coupled and coordinated development of the dual chains and firm innovation investment

Innovation investment constitutes a critical component in firms’ efforts to pursue technological advancement and product upgrading, and it generally refers to the allocation of resources, such as capital, talent, and R&D activities, aimed at enhancing firms’ innovation capacity (44). Ensuring sustained investment in innovation is essential for firms to remain competitive and responsive in dynamic market environments. When firms actively assume the central role in driving technological innovation, they contribute to the formation of a firm-level innovation ecosystem, which significantly improves the quality and value of innovation outcomes (45). In the TCM industry, this innovation imperative is increasingly shaped by the convergence of ancient empirical knowledge and cutting-edge technologies, with artificial intelligence emerging as a transformative force in enhancing diagnostic precision and accelerating therapeutic development (46).

From the perspective of talent, such an innovation ecosystem strengthens a firm’s ability to attract and retain diverse types of innovation-oriented personnel. As shown in the findings of Sun and Wei (47), firms that develop robust science and innovation systems are more effective at drawing in high-quality human capital, which in turn supports the implementation of innovation activities and facilitates sustained innovation investment. Talent, as a core element in firm development, not only fosters the effective execution of innovation projects but also contributes to the creation of a favorable internal innovation climate, laying the foundation for long-term development.

From the supply chain perspective, the integration of innovation systems with supply chain operations enhances the configuration of internal and external resources and significantly increases innovation efficiency. This enables firms to conduct innovation activities more effectively, reduce innovation cycles, and respond more rapidly to market demands. At the same time, research by Sun et al. (48) points out that as supply chain concentration increases, so does the exposure to supply-side risks, which often compels firms to intensify their innovation efforts in order to increase product differentiation and sustain their competitive advantage. Taken together, the coupled and coordinated development of the talent chain and supply chain not only enhances firms’ innovation capabilities and market competitiveness, providing crucial support for long-term development, but also plays a pivotal role in shaping the scale and direction of innovation investment. Based on this, the following hypothesis is proposed:

*H4*: The coupled and coordinated development of the talent chain and supply chain significantly promotes firms’ innovation investment.

### 2.5 The mechanism through which dual-chain coupling and coordination influence new quality productive forces

The tight coupling and coordinated development between the talent chain and the supply chain establish an efficient and integrated management mechanism that plays a critical role in the advancement of firms. This mechanism is particularly effective in facilitating technological upgrading, as it significantly enhances firms’ motivation for R&D investment and, under the influence of market forces, promotes synergy and complementarity among various innovation factors, thereby improving resource allocation efficiency and ultimately advancing the development of new quality productive forces (49). Prior research suggests that under such a synergistic framework, key personnel, including decision-makers, managers, and technical experts, play a pivotal role in driving technological innovation. Their deep domain knowledge and acute market sensitivity enable them to steer firms toward innovation-oriented strategies, especially those associated with exploratory innovation, which often yields more transformative outcomes (50). At the same time, the supply chain, as the front-end interface connecting firms to markets, offers an essential feedback mechanism that grounds R&D activities in real-time market demand, thus greatly enhancing the precision and effectiveness of innovation efforts.

For startups and small and medium-sized enterprises operating in dynamic environments, this dual-chain coupling mechanism enables them to respond more flexibly to external changes by integrating supply chain resources, identifying emerging opportunities, and fostering innovation and growth. Notably, this mechanism contributes not only to the sustained growth of R&D investment but also to a marked improvement in firms’ technological innovation capabilities (51). Driven by increased innovation input, firms are able to optimize their product portfolios and significantly boost production efficiency, which in turn accelerates the development of new quality productive forces and strengthens core competitiveness. Moreover, the coupled coordination between the talent and supply chains enhances firms’ sustainability. Through sustained innovation activities, firms can develop environmentally friendly, energy-efficient, and high-performance products and technologies, aligning with contemporary market trends while fulfilling broader social responsibilities (42). In summary, the synergistic interaction between the talent chain and the supply chain promotes increased innovation investment, which serves as a powerful driver for the development of new quality productive forces, while also reinforcing firms’ capacity for sustained innovation and long-term competitiveness. Based on this, the following hypothesis is proposed:

*H5*: the coupled and coordinated development of the talent chain and the supply chain influences the development of new quality productive forces by promoting firms’ innovation investment.

**FIGURE 1:**
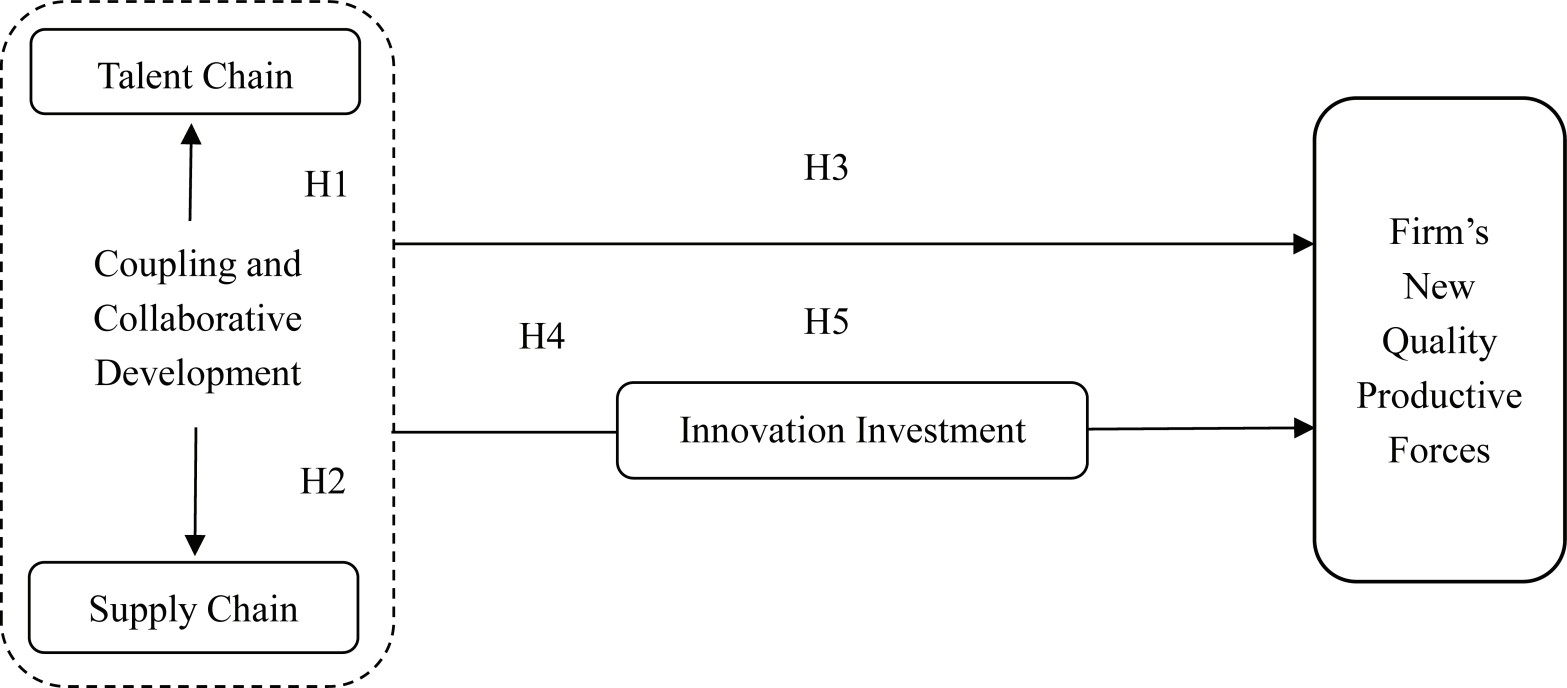
Theoretical model.

Accordingly, this study proposes a framework (Figure 1) in which the coupled and coordinated development of the talent and supply chains generates synergistic effects that shape firms’ NQPF. This dual-chain interaction is posited to enhance innovation investment, which in turn may support NQPF improvement. By emphasizing the interplay between resource integration and innovation dynamics, the model offers a process-oriented perspective on how coordinated capabilities contribute to NQPF.

## 3 The modeling and data issues

### 3.1 Samples and data sources

This study focuses on 75 TCM manufacturing enterprises listed on the A-share market and the New Third Board between 2015 and 2023. Considering that the period after 2015 represents a phase of rapid technological advancement in China, as well as the specific sample requirements for leading firms and data availability, the selected sample covers all Chinese medicine manufacturers listed on the Shenzhen Stock Exchange, the Hong Kong Stock Exchange, and the Shanghai Stock Exchange, and conforms to the definition of leading enterprises in the Chinese medicine supply chain. Based on the initial sample, observations with missing or anomalous values in relevant variables were excluded. For certain firms with incomplete data in individual years, linear interpolation was applied to fill in the missing values before conducting the analysis. Relevant data were primarily sourced from the CSMAR and CNRDS databases, as well as from corporate annual reports. Detailed information on the specific indicators is provided in Tables 1, 2.

**TABLE 1:** Indicators of the NQPF for the TCM manufacturing industry.

Target level	Criterion level	Primary indicator	Secondary indicator	Measurement	Indicator description	Data source
**N** **Q** **P** **F**	Labor force	Executive capital	Chairman’s human capital	Chairman’s educational attainment	PhD = 5, Master = 4, Bachelor = 3, Associate = 2, High school or below = 1	CSMAR, annual reports
			Executive team human capital	Average educational attainment of executive team		
		Employee quality	R&D personnel	Proportion of R&D personnel	Number of R&D staff/total number of employees	
			Highly educated staff	Proportion of employees with postgraduate degrees or above	Number of employees with postgraduate degrees or above/Total number of employees	
	Objects of labor	Ecological environment	Environmental performance	Environmental rating	ESG ratings from AAA to NA, converted to values from 1 to 7	Chengxin ESG rating
		Manufacturing expense	Manufacturing cost	Manufacturing expense ratio	(Total cash outflows from operating activities + depreciation + amortization + impairment provision − payments for goods/services − employee wages)/(Total cash outflows from operating activities + depreciation + amortization + impairment provision)	CSMAR, annual reports
	Means of labor	Technological means of labor	Technological innovation level	Entropy-weight result of R&D intensity, patents, and intangible asset proportion	Total R&D expenditure/operating revenue Number of patents Intangible assets/total assets	CNRDS, CSMAR
		Digital means of labor	Digitalization level	Entropy-weighted results of digital technology-driven and digital application indices	Scores of the digital transformation index Score of the digital application	CSMAR
		Traditional means of labor	Fixed Asset investment	Proportion of fixed assets	Fixed assets/total assets	

**TABLE 2:** Variable definition.

Variable type	Variables	Measurement	Data source
Dependent variable	NQPF	Composite index covering laborers, labor objects, and labor instruments	Constructed by author
Independent variables	Talent chain	Composite score reflecting the overall quality of decision-making, managerial, and technical personnel	
	Supply chain	Composite score based on supplier relationships, customer preparedness, and client dependency	
	Dual-chain coupling	Coupling coordination degree between talent chain and supply chain	
Mediating variable	Innovation investment	Composite index reflecting both R&D expenditure and innovation-related human resource investment	
Control variables	Employees	Total number of firm employees	CSMAR
	Firm age	Number of years since the firm’s establishment	
	Asset-liability ratio	Total liabilities/total assets	
	Revenue growth rate	(Current year revenue - previous year revenue)/Previous year revenue	
	Net operating cash flow	Net cash inflow or outflow from operating activities	
	Ownership concentration	Shareholding ratio of the top ten shareholders	
	CEO duality	Equals 1 if the chairman and general manager positions are held by the same person, 0 otherwise	
	Managerial ownership ratio	Shares held by directors, supervisors, and executives/total shares	
	Board size	ln(Number of board members + 1)	
	Independent director ratio	Number of independent directors/total number of board members	

The NQPF index is constructed using the entropy weight method based on 12 indicators across three dimensions (laborers, objects of labor, and means of labor), with final scores multiplied by 100 for ease of interpretation (see Table 1 for detailed indicators). The talent chain index is similarly derived via entropy weighting from indicators at the decision-making, managerial, and technical levels. Innovation investment is a composite measure that incorporates both R&D expenditure intensity (R&D expenditure/operating revenue) and the proportion of R&D personnel, capturing both financial and human capital dimensions of innovation input.

### 3.2 Construction of a new quality of productive forces in enterprises

NQPF are characterized by technological innovation, driven by high-quality development, and fundamentally reflected in the relative improvement and optimization of productivity (52). As a dynamic and gradually transformative form of productivity, they represent a new productive paradigm powered by advanced R&D and management teams, modern production tools, and technical systems (16). In the context of TCM manufacturing, NQPF are centered on breakthroughs in key and disruptive technologies, and involve a substantial upgrade in the combination of laborers, means of labor, and objects of labor.

Laborers are the most dynamic component of productivity, and their quality and capabilities directly determine the development level of NQPF in the TCM manufacturing sector (53). In this dimension, we further distinguish between the quality of the decision-making level and that of general employees. When exploring NQPF, firms encounter various challenges, and board chairpersons, as the primary agents of innovation, play a crucial role in technological advancement, talent development, and policy alignment. Moreover, managerial personnel, who serve as the backbone of firm operations and are skilled in applying advanced production resources, contribute significantly to the formation of NQPF. Accordingly, the quality of the decision-making level is reflected through the human capital of board chairs and senior executives, measured by their educational attainment. In parallel, the quality of general employees, particularly R&D staff, serves as the foundation of innovation in TCM firms. The professional competence, creativity, and experience of these employees directly affect the pace and quality of new drug development, which is why indicators such as the proportion of R&D personnel and those holding postgraduate degrees are taken into account.

As the material foundation of productivity (52), the objects of labor also play a vital role in the TCM manufacturing industry. In this regard, we assess two aspects: the ecological environment and manufacturing expenses. Under the framework of NQPF, enterprises are expected not only to pursue economic gains but also to prioritize environmental protection, corporate social responsibility, and governance. Environmental performance is evaluated using the environmental rating from the China Chengxin Green Finance ESG scoring system (54), while manufacturing expenses reflect how efficiently TCM manufacturers utilize raw materials and energy during the production process. Enhancing production processes and improving efficiency allow enterprises to reduce manufacturing costs, thereby contributing to the advancement of NQPF.

The means of labor, serving as the technical instruments of productivity (55), are also central to the development of NQPF in TCM manufacturing. As digital technologies and artificial intelligence become more widely adopted, firms are increasingly moving toward intelligent, automated, and refined production systems. The integration of such technologies not only raises productivity but also lowers production costs, offering robust support for the high-quality development of the sector. Therefore, this study incorporates indicators such as technological innovation level, digitalization level, and fixed asset investment to capture enterprise efforts and achievements regarding the means of labor.

Based on the conceptual framework of Zhiguang et al. (56) and adhering to the principles of scientific rigor, feasibility, measurability, and data availability, we construct an evaluation index system for the development level of NQPF in TCM manufacturing. Since the initially measured NQPF index ranges between 0 and 1, we multiplied it by 100 to facilitate subsequent empirical analysis. This system in Table 1 is organized around the three core dimensions of laborers, objects of labor, and means of labor, and comprises 7 first-level indicators and 12 specific measurement indicators.

### 3.3 Model specification and variable definitions

#### 3.3.1 Dependent variable

##### 3.3.1.1 NQPF of enterprises

Based on the conceptual definition of NQPF in the context of the TCM manufacturing industry and drawing on the methodologies (21, 57), this paper constructs an evaluation index system from three key dimensions: laborers, objects of labor, and means of labor. The entropy weight method is employed to determine the weights of indicators at each hierarchical level.

#### 3.3.2 Independent variables

(1) Talent chain: Following the approach (51), this study constructs a multi-dimensional index system based on decision-making, managerial, and technical levels, with weights determined using the entropy method.

(2) Supply chain: Enterprise supply chain resilience refers to the capability of firms to maintain continuity, recover quickly, and adapt effectively in the face of external shocks. Based on Zhou et al. (58), this study evaluates the supply chain level of TCM manufacturers using the entropy weight method across supplier reliability, customer preparedness, and supply dependency.

(3) Dual-chain coupled synergy: This refers to the degree of coupling and coordination between the talent chain and the supply chain, capturing the synergistic effect of human resource and supply configuration. The strength of synergy is measured through a coupling coordination degree model between the talent chain and the supply chain. The specific model is as follows:


$$C={\left[\frac{\prod_{i=1}^{n}U_{i}}{\left(\frac{1}{n}\,\sum_{i=1}^{n}U_{i}\right)}\right]}^{\frac{1}{n}}$$

In the formula, *n* denotes the number of subsystems, *U_i_* represents the value of each subsystem, and *C* stands for the coupling degree, where a higher value indicates a stronger coupling interaction. Since this study involves the talent chain and the supply chain, so *n* is set to 2. Accordingly, the coupling coordination degree model is as follows:


$$D=\sqrt{C\times T}$$

$$T=\alpha \,U_{1}+\beta \,U_{2}$$

In the formula, *D* represents the coupling coordination degree, and *T* denotes the comprehensive coordination index of the talent chain and supply chain. α *and*β are the weights assigned to each subsystem. As this study considers the talent chain and supply chain to be equally important, both α and β are set to 0.5. The classification criteria for coupling coordination degree are clearly defined. The coupling coordination degree is divided into several intervals, each corresponding to a specific level of coordination. These levels range from severe imbalance (0–0.1) to high-quality coordination (0.9–1.0), and include categories such as high, moderate, and mild imbalance, as well as on the verge of imbalance, barely coordinated, primary coordination, intermediate coordination, and good coordination.

#### 3.3.3 Mediator variable

##### 3.3.3.1 Innovation Investment

Drawing on the findings (59), innovation input reflects a firm’s capacity and willingness to generate internal knowledge and absorb external knowledge. This study captures innovation input through a composite measure that includes both financial and human capital investment in innovation, represented by R&D expenditure and innovation-related human resource input.

#### 3.3.4 Control variables

Following Ma et al. (60), this study includes the following firm-level control variables: number of employees, firm age, asset-liability ratio, revenue growth rate, net operating cash flow, ownership concentration, CEO duality, managerial ownership ratio, board size, and the proportion of independent directors. To examine the mediating effect of innovation input, the study adopts a stepwise regression method, comprising three stages: (1) Testing the effect of the coupling coordination of the dual chains on enterprise NQPF; (2) Assessing the relationship between the coupling coordination of the dual chains and innovation investment; (3) Examining the joint effects of dual-chain coupling coordination and innovation investment on NQPF.

### 3.4 Descriptive statistics and correlation test

The descriptive statistics of the main variables are presented in Table 3. Regarding NQPF, the values range from 19.61 to 51.44, with a mean of 30.68 and a standard deviation of 5.445. This distribution indicates a certain level of heterogeneity in NQPF across TCM manufacturing enterprises. While the degree of dispersion among firms is relatively small, there remains considerable room for improvement in overall productivity. For the talent chain, values range from 0.087 to 0.866, with a mean of 0.367 and a standard deviation of 0.104.

**TABLE 3:** Variable descriptive statistical results.

Variables	N	Mean	p50	SD	Min	Max	VIF
NQPF	556	30.68	30.08	5.445	19.61	51.44	–
Talent chain	556	0.367	0.358	0.104	0.087	0.866	2.720
Supply chain	556	0.073	0.049	0.075	0.012	0.636	9.520
Dual-chain coupling	556	0.381	0.368	0.071	0.202	0.673	10.18
Employees	603	3.882	2.353	4.444	0.106	28.05	3.290
Firm Age	648	21.72	22	5.916	6	43	1.270
Asset-liability ratio	624	0.390	0.305	1.629	0	40.78	1.300
Revenue growth rate	616	0.118	0.095	0.508	−0.859	11.33	1.050
Net operating cash flow	629	0.486	0.172	0.884	−1.120	6.999	2.800
Ownership concentration	585	0.596	0.604	0.167	0.214	0.992	1.310
CEO duality	666	0.327	0	0.470	0	1	1.160
Managerial ownership ratio	436	0.184	0.111	0.198	0	0.947	1.100
Board size	570	8.516	9	2.024	1	16	1.970
Independent director ratio	549	0.367	0.333	0.071	0	0.660	1.640

This suggests that while some firms have made notable progress in talent chain development, the majority still lag behind, with a pronounced gap between enterprises. The supply chain shows values ranging from 0.012 to 0.636, with a mean of 0.073 and a standard deviation of 0.075, indicating substantial variation in supply chain development across firms. In terms of the dual chain coupling, the maximum value is 0.673, the minimum is 0.202, and the mean is 0.381. This suggests that the overall coupling coordination between the talent chain and supply chain among listed TCM firms is relatively low, with most firms experiencing mild misalignment. However, the standard deviation of 0.071 indicates a relatively balanced distribution of coupling coordination levels across firms. Additionally, results from the variance inflation factor (VIF) test show that, except for the dual-chain coupling coordination degree, all other variables have VIF values well below 10. This confirms that multicollinearity is not a concern in this study, thereby ensuring the reliability of the subsequent regression analysis.

## 4 Empirical results

### 4.1 Benchmark regression analysis

Through the Hausman test for model specification discrimination, the results indicate that a two-way fixed effects model (controlling for both firm and year effects) represents the optimal choice for the baseline regression. Table 4 presents the baseline regression results of this study. Column (1) focuses on the relationship between the talent chain and firms’ NQPF. The regression coefficient for the talent chain is 29.463 and is statistically significant at the 1% level, indicating a robust positive effect of the talent chain on the NQPF of leading firms in the TCM manufacturing sector. This suggests that strengthening and optimizing the talent chain can effectively enhance firms’ NQPF, thereby providing a strong human capital foundation for sustained innovation and development.

**TABLE 4:** Benchmark regression results.

Variables	NQPF			
	(1)	(2)	(3)	(4)
Talent chain	29.463***	30.737***	31.016***	
	(2.042)	(2.854)	(4.046)	
Supply chain				5.179*
				(2.708)
Employees		0.001	0.001	−0.003**
		(0.001)	(0.001)	(0.001)
Age		0.039	−0.185***	−0.096
		(0.052)	(0.064)	(0.068)
Asset-liability ratio		−1.139	−1.100	0.229
		(1.207)	(2.342)	(2.537)
Revenue growth rate		−1.097**	−1.082	−1.272
		(0.458)	(0.876)	(0.970)
Net operating cash flow		−0.002	−0.003	−0.002
		(0.002)	(0.002)	(0.003)
Ownership concentration		0.292	1.596	3.230
		(1.959)	(3.076)	(3.702)
CEO duality		−0.229	−0.275	−0.751
		(0.353)	(0.488)	(0.592)
Managerial ownership ratio		1.002	0.871	1.136
		(0.840)	(1.150)	(1.383)
Board size		0.051	0.075	0.102
		(0.143)	(0.211)	(0.300)
Independent director ratio		2.434	3.015	3.092
		(3.242)	(5.073)	(5.575)
Year	No	No	Yes	Yes
_cons	19.871***	17.367***	20.641***	28.870***
	(0.754)	(2.941)	(4.503)	(5.567)
*N*	556.000	401.000	401.000	401.000
*R* ^2^	0.299	0.335	0.353	0.131
*R*^2^_a	0.204	0.216	0.321	0.087

Standard errors in parentheses,

*p < 0.1,

**p < 0.05,

***p < 0.01.

Column (2) incorporates control variables to further examine the impact of the talent chain on NQPF. The *R*^2^ increases to 0.335, indicating an improved explanatory power of the model. The coefficient of the talent chain remains statistically significant, providing additional evidence for its positive effect. To more accurately account for potential time-related disturbances during the sample period, a fixed-effects model is applied to control for year-specific influences. Following this adjustment, the *R*^2^ increases further, and the positive relationship between the talent chain and NQPF remains significant, suggesting that the estimated effect is robust even when accounting for time-fixed effects.

Column (4) examines the relationship between the supply chain and NQPF. The regression coefficient for the supply chain is 5.179 and is significant at the 10% level, indicating that supply chain development also plays a positive and statistically significant role in enhancing firms’ NQPF. This finding highlights the critical importance of effective supply chain management and coordination in strengthening firms’ competitiveness and innovation capabilities. Overall, the baseline regression results reported in Table 4 provide empirical support for Hypotheses H1 and H2, affirming that both the talent chain and the supply chain exert significant positive influences on the development of NQPF in leading firms in the TCM industry.

### 4.2 Mediation effect analysis

Table 5 presents the regression results for the mediation effect analysis. In Column (1), the focus is placed on the direct impact of dual-chain coupling coordination on firms’ NQPF. The regression results show that the coefficient for dual-chain coupling coordination is 19.136 and statistically significant at the 1% level, indicating that the coordination between the talent chain and the supply chain significantly enhances the development of NQPF, thereby supporting Hypothesis H3.

**TABLE 5:** Analysis of mediating effects.

Variables	NQPF	Innovation investment	NQPF
	(1)	(2)	(3)
Dual-chain coupling	19.136***	23.446*	14.106**
	(5.955)	(12.163)	(6.825)
Innovation investment			0.258***
			(0.080)
Employees	−0.004***	−0.426	−0.001
	(0.001)	(0.391)	(0.002)
Age	−0.064	−0.230	0.274***
	(0.067)	(0.213)	(0.094)
Asset-liability ratio	−0.013	1.380	−1.388
	(2.547)	(3.935)	(1.601)
Revenue growth Rate	−1.379	−2.049**	−0.641
	(0.913)	(0.880)	(0.639)
Net operating cash flow	−0.004	−0.780**	−0.001
	(0.003)	(0.361)	(0.002)
Ownership concentration	3.850	4.160	0.452
	(3.218)	(4.599)	(3.197)
CEO duality	−0.836	−1.411*	−0.184
	(0.563)	(0.726)	(0.518)
Managerial ownership ratio	1.513	4.135**	0.347
	(1.164)	(1.770)	(0.997)
Board Size	0.105	0.805	−0.149
	(0.280)	(0.670)	(0.343)
Independent Director Ratio	3.583	14.289	2.911
	(5.340)	(12.127)	(8.157)
Year	Yes	Yes	Yes
_cons	21.453***	−2.852	16.090***
	(5.270)	(10.002)	(5.727)
*N*	401.000	309.000	309.000
*R* ^2^	0.177	0.188	0.357
*R*^2^_a	0.136	0.140	0.317

Standard errors in parentheses,

*p < 0.1,

**p < 0.05,

***p < 0.01.

Column (2) further examines the relationship between dual-chain coupling coordination and firms’ innovation input, serving as the first stage of the mediation analysis. The results indicate that the regression coefficient of dual-chain coupling coordination on innovation input is 23.446 and significant at the 10% level, suggesting that dual-chain coordination significantly promotes innovation investment, thereby validating Hypothesis H4. Column (3) introduces innovation input into the regression model based on Column (1), in order to assess whether it plays a mediating role in the relationship between dual-chain coordination and NQPF. The analysis reveals that innovation input exerts a positive and statistically significant influence, with a coefficient of 0.258 at the 1% significance level. Simultaneously, the coefficient for dual-chain coupling coordination remains significant at the 5% level, with a reduced value of 14.106, which further confirms the partial mediation effect of innovation input in this relationship. To verify the robustness of the mediation effect, the *Sobel* test is conducted, and the results are significant at the 1% level. The mediation effect accounts for 26.29% of the total effect, indicating that innovation input plays a substantial bridging role in the impact of dual-chain coupling coordination on NQPF. In summary, the findings reported in Table 5 provide clear evidence that dual-chain coupling coordination not only directly contributes to the enhancement of firms’ NQPF but also exerts a significant indirect effect through the mediating role of innovation input, thus lending support to Hypothesis H5.

### 4.3 Robustness checks

Technological innovation and the optimization of resource allocation efficiency constitute the primary driving forces behind significant improvements in total factor productivity (TFP), and they also represent a critical pathway for cultivating new quality productive forces. Although various approaches to measuring productivity exist in the academic literature, the Solow residual method remains the most widely adopted and operationalizable metric for calculating TFP. At the micro-enterprise level, the Cobb-Douglas production function is commonly used to model the relationship between input factors such as capital and labor and the resulting output. In this context, TFP is treated as the residual component that cannot be directly explained by capital and labor, thereby capturing the combined effects of technological progress, managerial innovation, and resource allocation efficiency. This understanding of TFP closely aligns with the conceptual connotation of NQPF, which encompasses both technical advancement and improvements in efficiency. Drawing on the study (61), the present paper adopts TFP as a proxy variable for NQPF and conducts robustness checks to assess its validity. Furthermore, following the methodology of Ding et al. (62), we apply multiple estimation approaches for TFP, including the Olley-Pakes (OP) method, the Levinsohn-Petrin (LP) method, and the Generalized Method of Moments (GMM), to ensure the robustness of our findings. In this study, both the OP and GMM methods are employed for robustness verification.

Table 6 presents the results of the robustness analysis. Columns (1)–(3) report the regression outcomes using the OP method, examining the effects of the talent chain, supply chain, and dual-chain coupling coordination on NQPF. The results indicate that the talent chain has a positive and significant coefficient of 0.982 at the 5% level, the supply chain exhibits a coefficient of 3.421 that is significant at the 1% level, and the dual-chain coupling coordination yields a coefficient of 4.272, which is also significant at the 1% level. These findings suggest that all three factors exert a significantly positive influence on new quality productive forces.

**TABLE 6:** Robustness test results.

Variables	TFP_OP			TFP_GMM		
	(1)	(2)	(3)	(4)	(5)	(6)
Talent chain	0.982**			0.967**		
	(0.375)			(0.378)		
Supply chain		3.421***			3.288***	
		(1.021)			(1.091)	
Dual-chain coupling			4.272***			4.169***
			(1.064)			(1.165)
Employees	0.005	−0.057***		0.012	−0.047**	
	(0.027)	(0.020)		(0.024)	(0.020)	
Age	0.007	0.007	0.013***	0.009	0.009	0.016***
	(0.005)	(0.006)	(0.005)	(0.006)	(0.006)	(0.005)
Asset-liability ratio	0.124	0.203	0.020	−0.023	0.053	−0.106
	(0.243)	(0.216)	(0.226)	(0.247)	(0.231)	(0.245)
Revenue growth rate	0.346***	0.344***	0.315***	0.369***	0.368***	0.340***
	(0.070)	(0.062)	(0.057)	(0.077)	(0.071)	(0.069)
Net operating cash flow	0.104***	0.029	0.020	0.097***	0.025	0.019
	(0.037)	(0.031)	(0.021)	(0.035)	(0.031)	(0.022)
Ownership concentration	−1.451**	−1.225**	−1.189**	−1.425*	−1.207**	−1.172**
	(0.720)	(0.556)	(0.518)	(0.741)	(0.573)	(0.532)
CEO duality	0.183***	0.117*	0.115**	0.176***	0.112*	0.113**
	(0.067)	(0.061)	(0.050)	(0.062)	(0.057)	(0.047)
Managerial ownership ratio	−0.096	−0.098	0.003	−0.084	−0.085	0.012
	(0.098)	(0.100)	(0.122)	(0.090)	(0.100)	(0.124)
Board size	0.028	0.027	0.029	0.031	0.030	0.032
	(0.023)	(0.021)	(0.023)	(0.023)	(0.020)	(0.023)
Independent director ratio	0.551	0.616	0.785*	0.374	0.436	0.583
	(0.480)	(0.468)	(0.441)	(0.450)	(0.429)	(0.406)
Year	Yes	Yes	Yes	Yes	Yes	Yes
_cons	0.856	1.050*	−0.678	0.590	0.783	−0.883**
	(0.679)	(0.562)	(0.440)	(0.668)	(0.549)	(0.430)
*N*	401.000	401.000	401.000	401.000	401.000	401.000
*R* ^2^	0.382	0.534	0.548	0.340	0.491	0.511
*R*^2^_a	0.352	0.511	0.526	0.307	0.466	0.488

Standard errors in parentheses,

*p < 0.1,

**p < 0.05,

***p < 0.01.

Columns (4)–(6) present the results based on the GMM estimation. The regression coefficients for the talent chain, supply chain, and dual-chain coupling coordination are 0.967, 3.288, and 4.169, respectively, all of which are statistically significant at the 5% level for the talent chain and at the 1% level for both the supply chain and dual-chain coordination. These outcomes further confirm that the positive impacts of the talent chain, supply chain, and their coordinated interaction remain robust across different estimation techniques. Taken together, the robustness checks presented in Table 6 demonstrate that when TFP, calculated via different methods, is employed as a proxy for new quality productive forces, the positive effects of the talent chain, supply chain, and dual-chain coupling coordination remain consistent and statistically robust. These findings provide strong empirical support for the core hypotheses.

### 4.4 Heterogeneity analysis

When analyzing the impact of dual-chain coupling coordination on the NQPF of TCM manufacturing enterprises, it is essential to account for differences in the external environments faced by firms across different regions and institutional contexts. Regional disparities in the TCM industry, such as variations in policy support, education levels, and healthcare services, may lead to heterogeneous productivity outcomes and innovation dynamics. Drawing on the findings from the Report on the Inheritance and Innovation Development of Regional Traditional Chinese Medicine, this study categorizes the sample firms into subgroups based on key TCM-related variables to examine whether the effects of dual-chain coupling coordination vary under different contextual conditions.

First, as shown in Table 7, regression results indicate that in regions characterized by high levels of TCM healthcare services, the coefficient of dual-chain coupling coordination on NQPF is 19.261 and is statistically significant at the 1% level. This finding suggests that in areas with more developed medical service systems, the positive effect of dual-chain coordination on enterprise NQPF is more pronounced. In contrast, for the low TCM healthcare services subgroup, the effect of dual-chain coupling on new quality productive forces is statistically insignificant. This may reflect constraints in resource allocation and market demand in less developed regions, which hinder the effective realization of dual-chain synergies.

**TABLE 7:** Heterogeneity test results.

Variables	NQPF					
	High TCM healthcare services	Low TCM healthcare services	High TCM education	Low TCM education	High TCM policy	Low TCM policy
Dual-chain coupling	19.261***	34.559	21.900***	−5.026	17.101***	28.072
	(4.657)	(21.154)	(4.890)	(11.316)	(4.539)	(21.688)
Employees	−0.004**	−0.005	−0.004***	0.002	−0.004***	0.015
	(0.002)	(0.007)	(0.002)	(0.011)	(0.001)	(0.012)
Age	0.214**	−0.058	−0.115	0.425	0.249***	0.324
	(0.084)	(0.400)	(0.260)	(0.313)	(0.080)	(0.421)
Asset-liability ratio	0.646	−1.073	0.472	8.621	0.114	6.372
	(1.698)	(5.417)	(1.858)	(5.612)	(1.554)	(6.168)
Revenue growth rate	−1.786***	2.130	−1.219**	−1.314	−1.029*	−4.976**
	(0.555)	(2.402)	(0.597)	(1.816)	(0.603)	(2.198)
Net operating cash flow	−0.005*	−0.003	−0.005*	0.001	−0.003	−0.004
	(0.003)	(0.010)	(0.003)	(0.016)	(0.003)	(0.012)
Ownership concentration	3.257	1.102	4.660*	6.553	2.449	6.393
	(2.695)	(11.082)	(2.703)	(10.824)	(2.592)	(10.469)
CEO duality	−0.812*	−1.658	−0.924**	0.037	−0.533	−1.156
	(0.438)	(1.731)	(0.435)	(1.503)	(0.448)	(1.990)
Managerial ownership ratio	1.645	4.917	2.004*	−7.274	1.797	1.859
	(0.998)	(9.324)	(1.185)	(5.333)	(1.139)	(2.886)
Board size	0.055	0.452	0.039	1.030*	−0.021	1.002*
	(0.179)	(1.115)	(0.182)	(0.524)	(0.192)	(0.528)
Independent director ratio	5.763	27.058	1.969	40.989***	1.544	24.915
	(4.153)	(21.898)	(4.146)	(14.471)	(4.128)	(15.320)
Year	Yes	Yes	Yes	Yes	Yes	Yes
_cons	16.348***	6.959	22.302***	−7.198	18.932***	−16.030
	(4.295)	(21.343)	(6.306)	(16.795)	(4.149)	(18.577)
*N*	320.000	81.000	315.000	86.000	317.000	84.000
*R* ^2^	0.182	0.211	0.210	0.289	0.177	0.337
*R*^2^_a	−0.039	−0.972	−0.012	−0.889	−0.048	−1.038

Standard errors in parentheses,

*p < 0.1,

**p < 0.05,

***p < 0.01.

Second, for the high TCM education level subgroup, the regression coefficient is 21.9 and also significant at the 1% level, indicating that regions with more advanced educational infrastructure in TCM foster stronger innovation capabilities and technical expertise. These conditions amplify the positive effect of dual-chain coupling coordination on NQPF. Conversely, in the low TCM education level subgroup, the coefficient is −5.026 and statistically insignificant, possibly suggesting that limited educational resources restrict the effectiveness of innovation inputs, thereby weakening the productivity-enhancing effects of dual-chain coupling.

Lastly, the results in Table 7 show that in regions with high levels of TCM policy support, the coefficient is 17.101 and significant at the 1% level. This implies that in areas with strong policy incentives and institutional support, the advantages of dual-chain coordination are reinforced by a favorable regulatory environment, which further enhances firms’ innovation motivation. By contrast, in the low policy support subgroup, although the coefficient reaches 28.072, it fails to achieve statistical significance. This may suggest that the uneven distribution of policy support reduces its effectiveness in directly boosting enterprise NQPF.

In summary, the heterogeneity analysis in Table 7 demonstrates that the impact of dual-chain coupling coordination on the NQPF of TCM manufacturing firms varies significantly across different regional and institutional contexts. Factors such as healthcare infrastructure, educational development, and policy support play a moderating role in this relationship, highlighting the importance of external environmental conditions in facilitating the transformation and upgrading of NQPF within the TCM industry.

## 5 Conclusion and implications

### 5.1 Conclusion

Focusing on leading firms in the TCM manufacturing sector, this study empirically investigates the impact of dual-chain (talent chain and supply chain) coupling coordination on the development of NQPF, using panel data from 75 publicly listed TCM enterprises during the period 2015–2023. Furthermore, the mediating role of innovation input is explored. The main conclusions are as follows: (1) The effective coupling and synergistic development of the talent chain and supply chain significantly enhance firms’ NQPF. (2) Innovation investment plays a critical mediating role between dual-chain synergy and the development of NQPF. (3) The positive effect of dual-chain coordination on NQPF is more pronounced in regions characterized by stronger TCM healthcare infrastructure, more developed TCM education systems, and more favorable policy environments.

### 5.2 Discussion

The findings of this study are consistent with existing literature and further enrich the understanding of how dual-chain coordination enhances firm-level productivity. Prior research has emphasized that technological progress and efficiency gains are key drivers of productivity growth (61, 62). This study contributes by elucidating how the integrated development of the talent and supply chains jointly supports the emergence of NQPF in the TCM industry.

The development of NQPF is not only contingent on technological innovation and resource reallocation, but also heavily dependent on the effective integration of the talent and supply chains. While previous studies have largely focused on the influence of single chains (33, 42), this research reveals the compound effect of dual-chain coordination. The synergy between talent and supply chains enhances organizational adaptability and competitiveness in complex market environments. Therefore, firms should emphasize dual-chain integration by optimizing talent structures and refining supply chain management, thus building a resilient and innovation-oriented system capable of responding to rapid market and technological changes.

To fully appreciate these synergistic effects, it is essential to examine the distinct characteristics of each chain. From the talent chain perspective, the unique features of the TCM industry significantly shape how dual-chain synergy is realized. The cultivation of TCM talent relies heavily on the master-apprentice mentorship model, an extended process wherein core professional competencies require prolonged practical accumulation. Consequently, synergy between talent and the supply chain involves more than mere positional alignment. It necessitates the bridging of specialized knowledge and the transmission of experiential insights. Personnel trained through this mentorship system possess an intimate familiarity with the properties of medicinal materials and processing techniques. When such experiential knowledge is integrated into supply chain operations, it enables more effective control over raw material quality and sharpens responsiveness to market demand. However, the lack of standardized criteria within the mentorship model has also engendered talent scarcity and uneven geographical distribution. Thus, the development of the talent chain in the TCM industry must prioritize the institutionalized integration of traditional knowledge inheritance with modern strategic human resource management.

Complementing the talent-side dynamics, the supply chain perspective reveals additional layers of sectoral specificity. The synergistic mechanisms of the TCM supply chain are marked by pronounced industry characteristics. The principle of Daodi (geo-authenticity) imposes stringent geographical constraints on medicinal material supply, as provenance is strictly circumscribed by natural conditions. This obligates enterprises to bear elevated costs and quality-related risks. Accordingly, synergy between the supply and talent chains must address not only efficiency improvements but also rigorous quality control and shared risk mitigation. The stable operation of the supply chain hinges on the assurance of raw material authenticity and batch-to-batch consistency—a requirement that finds direct complementarity with the specialized human capital embedded in the talent chain. Yet, the provenance limitations of Daodi materials also result in attenuated supply elasticity and heightened operational costs. Consequently, the construction of TCM supply chains must strike a deliberate balance between efficiency optimization and the prevention and control of quality risks.

Further examining the mechanisms at play, innovation investment emerges as both a direct driver and a catalyst within the dual-chain coordination framework. The study finds that the coupling of the talent and supply chains positively stimulates innovation investment, which further contributes to NQPF. Increased R&D spending and enhanced technological innovation enable firms to optimize product portfolios and improve operational efficiency (51). Innovation input encompasses not only financial investment but also the pursuit and application of new technologies and processes. In today’s highly competitive environment, enhancing R&D capabilities and fostering interdisciplinary technological integration are essential for achieving industrial upgrading and breakthrough innovation.

Finally, the broader ecosystem in which firms operate exerts a powerful moderating influence. Regional policy support, healthcare infrastructure, and educational resources play a critical role in shaping NQPF. A favorable policy environment and access to high-quality educational resources provide a solid foundation for innovation, particularly in the culturally embedded and innovation-intensive TCM sector. Enhancing the innovation ecosystem and leveraging regional advantages are vital for improving the competitiveness of the TCM industry (63, 64). Moreover, the development of TCM healthcare services and widespread TCM education contribute to talent development and market expansion, further propelling the industry’s innovation and growth.

### 5.3 Research limitations and future prospects

Although this study has yielded meaningful findings, certain limitations remain regarding sample scope, endogeneity treatment, and variable measurement, which warrant further refinement in future research. Specifically, the limitations and corresponding directions for future inquiry are outlined in the following four aspects.

Firstly, this study focuses exclusively on A-share listed Traditional Chinese Medicine manufacturing enterprises in China. The sample size is relatively constrained, and the analysis does not encompass unlisted small and medium-sized enterprises or other entities situated upstream and downstream along the TCM industrial chain. To a certain extent, this may affect the external validity and generalizability of the conclusions. Future research could beneficially expand the sample coverage to examine the applicability of the findings within a broader range of contexts.

Secondly, although we have conducted robustness checks employing techniques such as lagging the explanatory variables and substituting alternative estimation methods, the difficulty in identifying fully appropriate exogenous instrumental variables has precluded the complete elimination of potential endogeneity biases arising from reverse causality or omitted variable issues. Subsequent research endeavors could seek to introduce more exogenous instrumental variables, such as policy shocks or geographical characteristics, to further enhance the reliability of causal inference.

Thirdly, the measurement of the talent chain and supply chain in this study is primarily based on firm-level financial and personnel data, which fails to fully capture the micro-level operational processes and dynamic evolutionary characteristics of industrial chain synergy. Moreover, although the proxy indicators for new quality productive forces have been validated through multiple methodological approaches, the richness of their conceptual connotation remains difficult to fully encompass within a single metric. Future investigations may consider integrating multi-source data to construct a more comprehensive and multidimensional measurement framework.

### 5.4 Implications

This study not only provides theoretical support for the relationship between dual-chain coordination and the emergence of NQPF, but also offers practical policy recommendations for TCM manufacturing enterprises and local governments: (1) TCM enterprises should promote deep integration between the talent chain and supply chain, optimizing human resource allocation while enhancing technological capabilities and managerial efficiency. At the same time, it is essential to improve supply chain flexibility and responsiveness to support innovation-driven productivity growth. (2) Firms should intensify their investment in technological innovation and R&D, strengthen core technological competencies, and cultivate competitive products to secure long-term market advantages. (3) Local governments should formulate supportive policies to optimize the external environment for TCM industrial innovation, thereby creating favorable conditions for firms to enhance their innovation capacity and productive performance.

## Data Availability

Publicly available datasets were analyzed in this study. This data can be found at: Please contact Zhiguang Li for data requests.
